# Pre‐exposure prophylaxis use in blood donors in England

**DOI:** 10.1111/vox.70232

**Published:** 2026-03-09

**Authors:** Jaid Debrah, Claire Reynolds, Victoria Maddox, Shannah Secret, Hatice Baklan, Alieu Amara, Laura Else, Peter Simmonds, Susan R. Brailsford, Saye Khoo, Heli Harvala

**Affiliations:** ^1^ Radcliffe Department of Medicine University of Oxford Oxford UK; ^2^ NHS Blood and Transplant/UK Health Security Agency Epidemiology Unit NHS Blood and Transplant London UK; ^3^ Microbiology Services NHS Blood and Transplant London UK; ^4^ Nuffield Department of Medicine University of Oxford Oxford UK; ^5^ Centre for Experimental Therapeutics, University of Liverpool Liverpool UK; ^6^ Institute of Biomedicine, Faculty of Medicine, University of Turku and Turku University Hospital Turku Finland

**Keywords:** blood donors, GBMSM, HIV, PrEP, syphilis, TTI

## Abstract

**Background and Objectives:**

The For the Assessment of Individualized Risk (FAIR) framework, introduced by NHS Blood and Transplant (NHSBT) in 2021, aims to reduce stigma and improve equity in blood donor selection, particularly for gay, bisexual and other men who have sex with men (GBMSM). While pre‐exposure prophylaxis (PrEP) is highly effective at preventing sexual transmission of human immunodeficiency virus, its declared use excludes individuals from blood donation. This study examined PrEP use among male blood donors with current or past syphilis in England to evaluate guideline compliance and implications for blood safety.

**Materials and Methods:**

Residual plasma samples from syphilis‐positive male blood donors collected in 2023 were tested for PrEP. These data were combined with two previous studies of syphilis‐positive donors conducted between July 2018 and June 2024, incorporating demographics and reported PrEP use.

**Results:**

The rate of syphilis‐positive blood donations increased from 4.09 to 10.32 per 100,000 donations between 2018 and 2024 (*p* = 0.048, Mann–Kendall trend test) with a rising proportion of past syphilis cases attributed to GBMSM (18%–37%; *p* = 0.004, Fisher's test, *p* = 0.001 Mann–Kendall test); 7.1% of syphilis‐positive blood samples from male blood donors tested positive for PrEP in 2023, indicating frequent non‐compliance with donation guidelines.

**Conclusion:**

Persistent PrEP use among syphilis‐positive donors since 2018 suggests gaps in donor education regarding eligibility. Targeted public health interventions, particularly for younger GBMSM, are needed to strengthen sexual health education, PrEP messaging and awareness of donation criteria. Further research into other infections associated with high‐risk sexual behaviour is warranted.

**Highlights**

- Since 2021, blood donation rules have changed in the United Kingdom, allowing more gay, bisexual and other men who have sex with men (GBMSM) to give blood. GBMSM are the predominant users of pre‐exposure prophylaxis (PrEP) in the country and disproportionately impacted by bacterial sexually transmitted infections.
- Previous studies have identified male blood donors in England who are positive for syphilis and PrEP.
- This more longitudinal assessment again identifies PrEP use in male blood donors in England with syphilis and suggests gaps in donor education regarding eligibility.

## INTRODUCTION

Human immunodeficiency virus (HIV) was discovered in the 1980s and, as it disproportionately impacted gay, bisexual and other men who have sex with men (GBMSM) in the West, most blood services at that time implemented a permanent ban on blood donations from GBMSM. In England, this was replaced by a 12‐month deferral for GBMSM in 2011, which was further reduced to 3 months in 2017, before the behaviour‐based Assessment of Individualized Donor Risk (FAIR) policy was implemented in 2021 [1]. With this, individuals who have had anal sex with a new partner or multiple partners, take part in chemsex activity or have been diagnosed with gonorrhoea within 3 months are not eligible to donate blood. The joint work between the NHS Blood and Transplant (NHSBT) and UK Health and Security Agency (UKHSA) monitoring the number of recent blood‐borne virus infections and transfusion‐transmitted infections (TTIs) following the implementation of FAIR guidelines has shown there was no immediate impact on blood safety [2]. However, surveillance of UK blood donors has shown ongoing use of undeclared pre‐exposure or post‐exposure prophylaxis (PrEP/PEP) among syphilis‐positive donors, highlighting the importance of continued monitoring [3, 4].

GBMSM are disproportionately affected by sexually transmitted infections (STIs), accounting for 76% of infectious syphilis diagnoses in England 2023 [5]. They also accounted for an estimated 7.5% (range 1.9–10.6) of global cases from pooled prevalence data between 2000 and 2020 [6]. An increase in the number of infectious syphilis diagnoses has been reported in the United Kingdom: a 165% rise was noted in diagnoses between 2009 and 2018 in the overall population and a 236% rise among GBMSM, the likely contributing factor being the increased transmission risk behaviour such as condomless sex and HIV sero‐adaptive behavioural strategies [7, 8, 9]. Although PrEP has contributed to the declining numbers of new HIV diagnoses especially among GBMSM [10], a 7% increase in new HIV diagnoses was still reported in this group between 2022 and 2023 (761–811) and 85.2% of candidates for PrEP in England in 2023 were GBMSM (94,026/110,338) [11]. In England, a combination of emtricitabine (FTC) and tenofovir (TDF) in a single pill has been provided for free as first‐line prophylaxis since March 2020 although injectables, such as cabotegravir (CAB) PrEP, may soon be available [12]. When taken as indicated, PrEP has shown to reduce the risk of contracting HIV sexually with 86%–92% efficacy [13, 14].

While syphilis is not a major blood safety concern in the United Kingdom, blood donations have been screened for *Treponema pallidum* antibodies since the 1940s [2] and individuals with a history of syphilis are ineligible to donate. Similarly, those who have recently stopped using PrEP/PEP are deferred for 3 months before they can donate. Suboptimal PrEP use can lead to breakthrough HIV infection with delayed seroconversion, resulting in a reduced effectiveness of HIV blood donation screening [15, 16, 17]. Furthermore, several studies have identified a subgroup of PrEP users with a high incidence of bacterial STIs [14, 17, 18]. PrEP use can be considered as a marker for potentially higher risk sexual behaviour, especially when excluding discordant couples, and is likely associated with ongoing sexual risk behaviour among GBMSMs [9, 19, 20, 21]. This is a potential contradiction between personal safety for blood donors and blood safety. Even when the undetectable equals untransmissible, U=U, for sexually transmitted HIV infections, there is a small but significant transmission risk of HIV via blood transfusion where even undetectable viral loads can transmit.

In the United Kingdom, both syphilis and HIV disproportionately affect GBMSM. Since PrEP is predominantly used by high‐risk GBMSM, we have assessed PrEP/PEP use among syphilis‐positive male blood donors in 2023, and compared detection frequencies with those reported in our previous studies from 2018 to 2021 [3, 4].

## MATERIALS AND METHODS

### Donation testing for HIV and syphilis infection

All blood donations in England were individually tested for *T. pallidum* antibodies using automated haemagglutination test (TPHA, Newmarket Biomedical Ltd.) on the Beckman Coulter PK7300 analyser. All screen repeat reactive samples were submitted for confirmatory testing, which included two different *T. pallidum* total antibody tests (Murex Diasorin and Trinity Captia, UK), Immunoglobulin M (IgM) antibodies (Trinity Captia, UK), TPHA titration (NewBio, Telford, UK), rapid plasma reagin test  titration (NewBio, Telford, UK) and line immunoassay (Inno‐Lia, FujireBio, Japan). If a confirmed syphilis‐positive blood donor has previously donated, their most recent archive sample is requested for repeat syphilis testing. Universal HIV screening included nucleic acid testing (NAT; Cobas Roche, Burgess Hill, UK) and combined HIV antigen–antibody test (HIV Ag/Ab; Abbott Architect). NHSBT performs HIV NAT in minipools of 24 donations, which gives a 95% limit of detection of 616 IU/mL for HIV RNA. All antibody‐reactive and/or HIV RNA‐positive samples were submitted for further confirmatory testing. Donors with confirmed positive donations were invited for a post‐test discussion to explain testing results and arrange suitable onward referral while also identifying potential routes of syphilis or HIV acquisition, respectively.

The syphilis infection was considered to have been acquired within 24 months and classified as ‘recent’ if the donation tested IgM‐positive without a history of syphilis, the donor had recent symptoms/clinical history indicative of infectious syphilis or tested negative on the most recent previous donation within 2 years. Similarly, HIV infection was considered ‘recent’ if the clinical history was consistent with acute infection, HIV antibody‐avidity was low (below 0.3) or the donor tested negative on their most recent previous donation within 12 months. Infection status of every donor was checked with the unit medical virology consultant.

### Data on blood donors with HIV and/or syphilis infection

Anonymous data on donor age, gender and most likely source of infection were provided for donors with markers for current or past syphilis and/or HIV identified in England between January 2018 and December 2024 by the joint NHSBT/UKHSA Epidemiology Unit. More detailed anonymized data, including their self‐reported ethnicity, country of birth and likely risk factors for infection, were obtained for male blood donors with markers of syphilis in England, 2023. The country of birth, the source and likely risk factors for infection were obtained from the post‐test discussion, which aims to inform donors about their syphilis results and the follow‐up required. This data are also used to determine whether they had been compliant.

All donors sign a consent form at the time of donation; this is based on the expectation they have read the donor information leaflet. NHSBT consent covers their information to be used for research which improves NHSBT's knowledge of the donor population. The study was approved by the Blood Supply Clinical Audit, Risk and Effectiveness Committee of NHSBT on 14 September 2021 and renewed in 2023 (BSCR21022, BRCR23001).

### Testing of residual plasma samples for TDF and FTC residuals

This study included a total of 84 anonymized residual plasma samples collected from male blood donors with markers of syphilis in England in 2023. While 110 samples were confirmed with markers of syphilis in 2023, only this subset of 84 was retrievable for further testing. For comparison, 150 anonymized blood donation samples were obtained from first‐time donors in 2023.

Plasma samples were individually tested for TDF and FTC concentration using high‐performance liquid chromatography coupled with mass spectrometry (LC–MS/MS) as previously described [3, 4]. The lower limit of quantification was 1 ng/mL for TDF and 5 ng/mL for FTC. Lack of detection of these analytes indicated that no PrEP was taken in the 5 days preceding donation. Plasma TDF concentrations >10 ng/mL would be consistent with PrEP administration within the previous 48–72 h; plasma TDF concentrations of 100 ng/mL have been detected in PrEP users 16 h post‐dose [22]. As TDF/FTC is also found in PEP or HIV treatment preparations, it should be noted that without further information from the donors, we would be unable to differentiate between them. Donors are asked not to donate if they have HIV or think they need a test for HIV.

## RESULTS

### Blood donors with HIV and/or syphilis in England, January 2018–December 2024

Since January 2018, 746 blood donors have been confirmed positive for syphilis (Table 1). Out of these, 80% (598) were male blood donors. The numbers and rates of blood donors with past or current syphilis have increased from 65, or 4.09 per 100,000 donations in 2018, to 154, or 10.32 per 100,000 donations in 2024 (*p* = 0.048 by Mann–Kendall trend test). The syphilis cases attributed to GBMSM have also increased from 68 pre‐FAIR (January 2018–June 2021) to 170 post‐FAIR (July 2021–December 2024: *p =* 0.004 by Fisher's exact test, *p* = 0.001 by Mann–Kendall trend test). Furthermore, the number of current infections acquired during the last 2 years has also increased from 23 pre‐FAIR to 54 post‐FAIR (*p* = 0.009; Mann–Kendall test).

**TABLE 1:** Blood donors with Human immunodeficiency virus (HIV) (*n* = 51) and/or syphilis (*n* = 746) infection in England, 2018–2024.

Year	2018	2019	2020	2021A	2021B	2022	2023	2024
				Jan–Jun	Jul–Dec			
Blood donations made	1,588,685	1,545,945	1,472,677	777,318	765,085	1,517,125	1,502,434	1,492,302
All syphilis infections	65	87	59	57	66	118	140	154
Per 100,000 donations	4.09	5.63	4.01	7.33	8.63	7.78	9.32	10.32
Men with syphilis	45	67	39	50	55	98	110	134
GBMSM with syphilis	12	16	15	25	29	38	46	57
GBMSM with recent syphilis <2 years	4	6	9	4	7	12	16	19
Non‐GBMSM men with syphilis	33	51	24	25	26	60	64	77
Non‐GBMSM donors with syphilis	53	71	44	32	37	80	94	97
All HIV infections	3	9	8	5	4	8	5	9
Per 100,000 donations	0.19	0.58	0.54	0.64	0.52	0.53	0.33	0.6
Men with HIV	2^a^	6	7	3	3	7	4^a^	6^a^
GBMSM with HIV	0	1	2	2	2	3	1^a^	4^b^
GBMSM with recent HIV < 2 years	0	0	1	0	0	1	0	2

Abbreviation: GBMSM, gay, bisexual and other men who have sex with men.

^a^ Co‐infection with syphilis: 1 in 2018, 2 in 2023, 1 in 2024.

^b^ One further blood donor diagnosed with HIV and reported as post‐donation information.

Over the full period from January 2018 to December 2024, 51 blood donors were identified with HIV infection—75% (38) male and 29% (15) GBMSM (Table 1). It should also be noted that one further HIV infection in a GBMSM blood donor was reported to us following their diagnosis; they had previously taken PrEP but had not continued it while engaging in a new relationship (data not shown). The number of HIV cases attributed to GBMSM also increased from 5 pre‐FAIR to 10 post‐FAIR (not statistically significant change), with the number of current infections acquired during the past year increasing from 1 to 3. In 2024, four out of the nine HIV cases were among GBMSM. Furthermore, there were four cases of HIV/syphilis co‐infection, one in 2018, two in 2023 and one in 2024.

### Characteristics of syphilis‐infected male blood donors and evidence of PrEP use in England, 2018–2023

**FIGURE 1:**
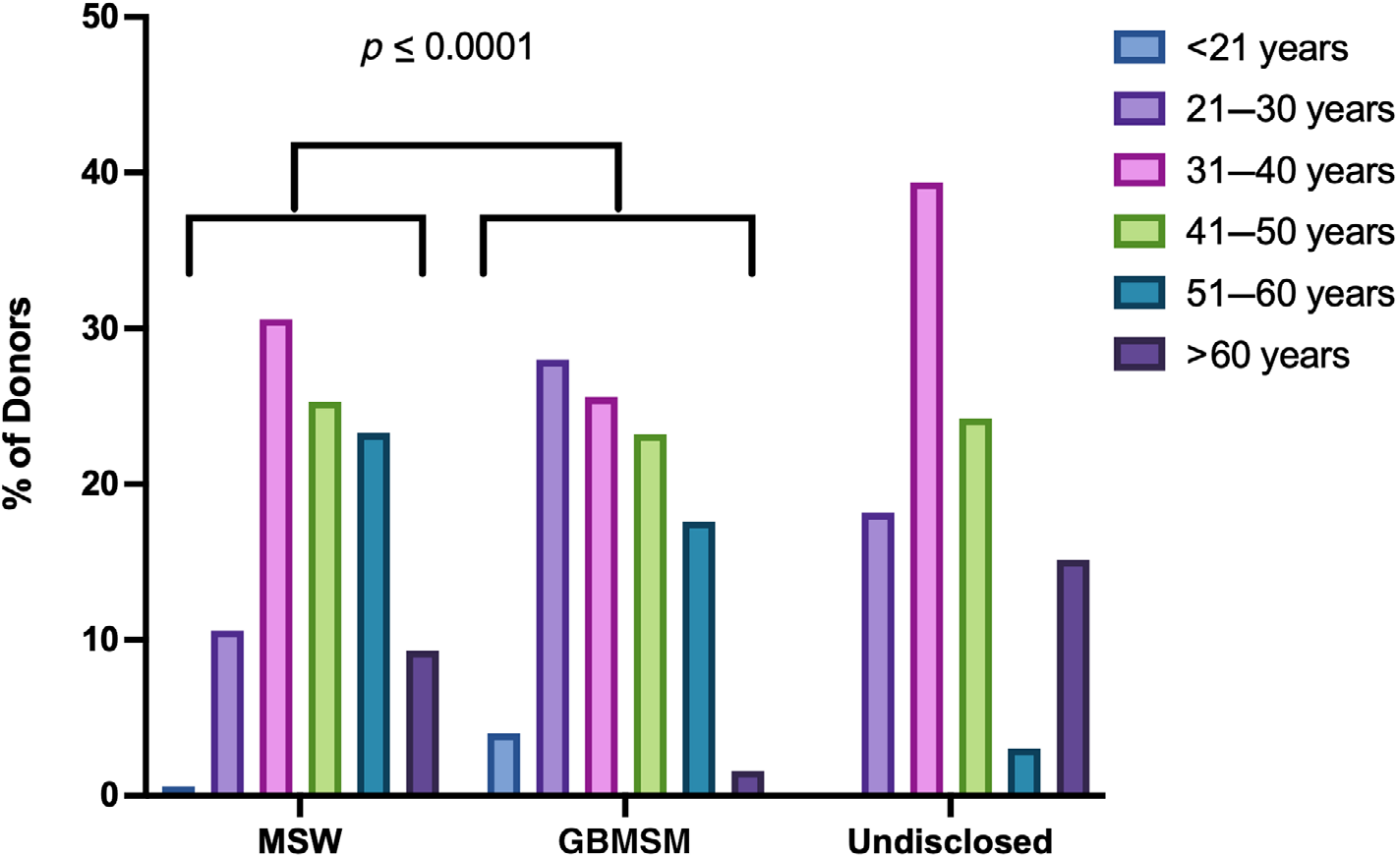
Proportion of syphilis‐positive male blood with female partners (*n* = 150), undisclosed partners (*n* = 33) and male donors with male partners (*n* = 125) by age group, England, 2018–2023. Mann–Whitney *U* test. MSW, men who have sex with women; GBMSM, gay, bisexual and other men who have sex with men.

Between 2018 and 2023, most male blood donors with syphilis who had declared female partners were between 31 and 60 years old (88.7%, 133/150), whereas most syphilis‐positive GBMSM donors were between 21 and 50 years old (80.8%, 101/125; Figure 1) (*p* < 0.0001, Mann–Whitney *U* test). Of donors who did not disclose their partner, most were between 21 and 50 years old (81.8%, 27/33). Only a small proportion of male blood donors with syphilis were below the age of 21 years (*n* = 6) or over 61 years (*n* = 21).

Six donation samples obtained from 84 male blood donors with syphilis identified in 2023 tested positive for both TDF and FTC, in comparison to 14 from 196 syphilis‐infected male donors between 2018 and 2022 (Table 2; data combined with published data from 2018 to 2022 [3, 4]). This is in contrast to testing of 150 randomly selected, syphilis‐negative new blood donor samples, which all tested negative for both TDF and FTC (6/84 vs. 0/150, *p* = 0.0019 by Fisher's exact test).

**TABLE 2:** Characteristics of syphilis‐positive male blood donors with recent pre‐exposure prophylaxis use identified in England, 2018–2023 (*n* = 20).

Year	Donor type	Age group	Ethnicity	UK born (yes/no)	Risk factors	Eligibility to donate based on post‐test discussion alone	Male partners (yes/no)	Syphilis status^b^	TDF (ng/mL)	FTC (ng/mL)	Inferred timing of PrEP
2018	First	51–60	Black African	N/A	Known past syphilis	No	No	Past	73.58	748.46	2–3 days
2019	Repeat	41–50	White British	N/A	Known past syphilis	No	Yes	Past	210.52	412.78	~1 day
2019	First	31–40	White Other	N/A	No response^a^	N/A	Yes	Past	39.96	ND	2–3 days
2020	First	31–40	White other	No	Same partner for 18 months	Yes	Yes	Past	<LLQ	10.33	<5 days
2020	First	19–21	White British	N/A	Male and female partners	Yes	Yes	Past	<LLQ	16.44	<5 days
2020	Repeat	41–50	White British	N/A	Known past syphilis	No	No	Past	37.76	74.83	2–3 days
2021	First	31–40	Southern American	No	Same partner for 2 years	Yes	Yes	Past	95.1	442.29	~1 day
2021	First	51–60	White British	Not asked	Known past syphilis	No	Yes	Past	43.61	532.77	2–3 days
2021	First	31–40	Southern American/Mixed	N/A	No response^a^	N/A	Not known	Past	59.7	686.14	2–3 days
2021	Repeat	41–50	White British	Yes	One‐off male partner	Yes	Yes	Past	73.97	136.59	2–3 days
2021	First	51–60	White British	N/A	Known past syphilis	No	Yes	Past	86.6	432.31	2–3 days
2021	Repeat	21–30	White British	N/A	No response^a^	N/A	Not known	Recent	7.3	33.36	<5 days
2021	First	41–50	White British	Yes	Known past syphilis	No	No	Past	8.82	18.66	<5 days
2022	First	41–50	White British	N/A	No response^a^	N/A	Not known	Past	84.91	544.76	2–3 days
2023	First	41–50	White British	Yes	Known past syphilis	No	Not known	Past	91.48	163.06	~1 day
2023	First	31–40	Asian Pakistani	N/A	Regular partner	Yes	Yes	Past	27.2	16.99	2–3 days
2023	First	51–60	White British	N/A	Known past syphilis	No	Yes	Past	15.97	7.65	2–3 days
2023	Repeat	51–60	White British	N/A	Occasional female partners	Yes	No	Past	69.29	615.65	2–3 days
2023	Repeat	21–30	Black Caribbean	N/A	Many new male partners	No	Yes	Past	89.45	1068.69	2–3 days
2023	First	31–40	Asian Pakistani	N/A	No response^a^	N/A	Not known	Past	22.79	73.5	2–3 days

Abbreviations: FTC, emtricitabine; PrEP, pre‐exposure prophylaxis; TDF, tenofovir; UK, United Kingdom; LLQ, detected but below the lower level of quantification.

^a^ N/A: no response to follow‐up questionnaire was received.

^b^ Infection was defined as recent if it was considered that the donor acquired the infection within 12 months before their last donation based on an Immunoglobulin M (IgM) ‐positive result or recent symptoms/clinical history indicative of infectious syphilis, or if they were negative on their most recent previous donation within 12 months.

**FIGURE 2:**
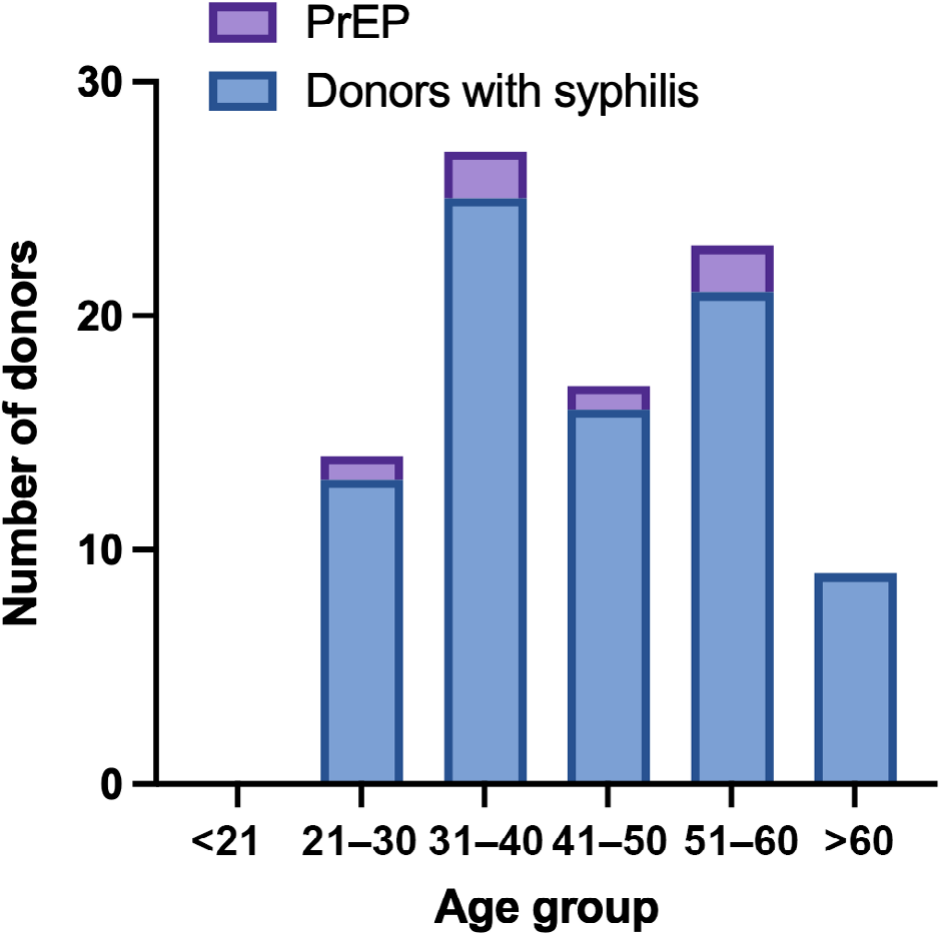
Number of syphilis‐positive male blood donors with or without pre‐exposure prophylaxis (PrEP) use, England 2023 (*n* = 84).

Six donors had evidence of recent PrEP usage; one had likely taken PrEP within a day from donation, and the remaining five within 2–3 days (Table 2). Three of these donors declared previous or current male sexual partners, one donor declared female partners only and two donors did not engage in a post‐test discussion. Half were of White British backgrounds (50%; 3/6), two were Asian Pakistani and one was of Black Caribbean heritage. Based on the post‐test discussions, three donors would not have been eligible to donate: one donor had not disclosed their multiple new male partners within 3 months of donation, and two other donors did not declare their previous syphilis diagnosis. The age ranged from 21 to 60 years (Figure 2).

**FIGURE 3:**
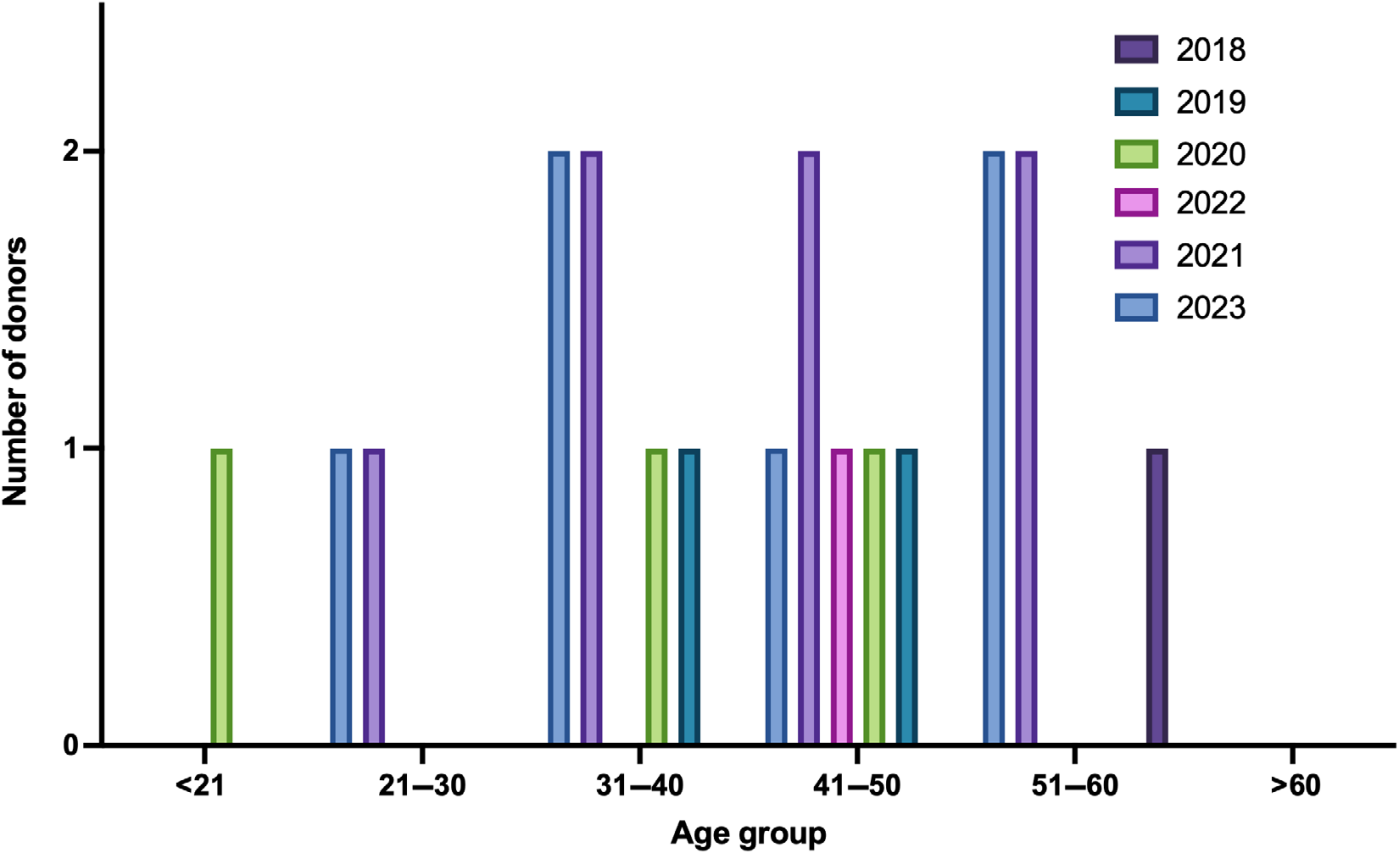
Number of syphilis‐positive male blood donors with pre‐exposure prophylaxis (PrEP) use by age and year of donation (*n* = 20).

The PrEP/PEP use among syphilis‐positive male blood donors including GBMSM has remained constant during the study period: it was 5.9% pre‐FAIR (10/169) and 7.1% post‐FAIR (10/141) (Figure 3). All except one donor with evidence of PrEP/PEP use had past syphilis (Table 2). Frequency of PrEP usage detected in donors was similar in different age ranges; 3 from the 60 samples tested from donors up to the age of 30 were PrEP positive compared to 17/247 from donors over 30 years old (*p* = 0.4 by Fisher's exact test). Furthermore, PrEP use among GBMSM with syphilis infections decreased from 12% pre‐FAIR (7/58) to 5.3% post‐FAIR (4/75). Syphilis‐infected donors with evidence of PrEP use identified during 2020–2023 were geographically dispersed (data not shown).

## DISCUSSION

This study describes the monitoring of PrEP/PEP use in male blood donors with syphilis infection in England following the implementation of FAIR. Although the number of blood donors with syphilis infections attributed to GBMSM has increased from pre‐FAIR, we have identified the persistent presence of PrEP/PEP use among syphilis‐positive male blood donors including GBMSM (4.3%, 20/464, between 2018 and 2023) [3, 4]. This was a higher percentage than in the Netherlands, where 2.6% of syphilis‐positive male donors showed evidence of PrEP use in 2020–2021 (1/39) [23]. We discuss the implications of these findings for blood services, donors themselves and public health policies.

Individuals with evidence of syphilis infection and PrEP/PEP use continue to donate blood in England. Since 2018, there has been a significant increase in both acute and past syphilis cases among blood donors. While this reflects a general increase in syphilis incidence in the United Kingdom, it is concerning that blood donors often did not disclose their history of syphilis, which would have excluded them from donation. Although past syphilis infection alone may not reflect recent high‐risk sexual behaviour of donors, both ongoing PrEP use and/or recent syphilis would argue for the opposite.

If PrEP is taken for need, it should be considered as a marker for potential sexual risk behaviour. A recent study from Denmark found that high‐risk sexual behaviour (i.e., condomless sex, chemsex) usually precedes PrEP initiation; it found a 35% increase in STI prevalence before donors started a PrEP regimen [21], indicating individuals sought PrEP/PEP as a result of high‐risk sexual activity. In England, PrEP is targeted towards those engaging in high‐risk sex, and 95% of PrEP users are GBMSM [24]. In our study, most syphilis‐infected blood donors with evidence on PrEP/PEP use had declared previous or current male partners (11/16, no data from the remaining four donors). The extent of PrEP/PEP use among blood donors without syphilis remains unknown in England, considering the small size of the control group included in this study. However, the identification of PrEP use among blood donors with syphilis in England despite the pre‐donation questionnaire presents a major gap in blood donor education, which must be prioritized. This might include, for example, advertisements, criteria reminder communications or even a digital shortened checklist before coming in to complete the pre‐donation questionnaire (a pre‐pre‐donation checklist) for a more immediate deferral.

Half of our PrEP users in 2023 were GBMSM; however, two donors did not disclose their sexual status. Non‐disclosure of history of sexual partners was also apparent in donors from the 2018–2021 studies, including in a PrEP user with an acute syphilis infection (Table 2). We know that 95% of PrEP users in the United Kingdom are GBMSM and, while PrEP/PEP use is not exclusive to GBMSM, this does raise questions about how comfortable donors are in disclosing their sexual history in pre‐ or post‐test discussions [24]. Fear of disclosure among lesbian, gay, bisexual and transgender/transsexual (LGBT+) individuals accessing health care has been well described in the literature, specifically the fear of being discriminated against, breaches in confidentiality (being forcibly outed) and/or receiving poor treatment because of disclosure [25, 26]. Indeed, 1 in 14 gay and lesbian individuals is expected to receive poorer treatment from healthcare staff because of their sexuality; 23% of LGBT+ individuals and 40% of bisexual men have described witnessing negative and discriminatory remarks made by healthcare professionals [27]. LGBT+ charity Stonewall has made recommendations to improve this mistrust through improved guidance and training for frontline staff. In the context of blood donation, this could optimize donation sessions and post‐screening discussions with first‐time GBMSM blood donors and potentially reduce the number of syphilis‐positive and/or PrEP users detected.

From the 20 syphilis‐positive male donors with evidence of PrEP use, most had declared previous or current male partners (11/15) and were aged between 31 and 60 years (17/20). PrEP usage in donors under 30 years old was not dissimilar to older donors. However, we did notice that the PrEP use among GBMSM had decreased since the introduction of FAIR. HIV incidence in young GBMSM is known to be higher, as well as suboptimal PrEP use, citing additional risks that can be summarized as a lack of education and experience [28, 29, 30]. This, combined with the five cases of newly identified HIV infections in young GBMSM blood donors since 2023—one of whom has a co‐infection with syphilis and one in a repeat donor who admitted after diagnosis that they had a new partner and had stopped taking PrEP—highlights the need for further public health interventions concerning education and PrEP distribution strategies. These should particularly target young GBMSM with the help of digital technologies, as well as continued collaboration with LGBT dating apps and youth charities [29].

Of the 34 syphilis‐positive GBMSM not using PrEP in 2023, less than half declared having one partner only and/or being in long‐term relationships (*n* = 16, 47%). While NHSBT donor criteria for GBMSM select those who have not used PrEP and have not had anal sex with a new sexual partner in the last 3 months, the number of syphilis‐infected GBMSM not eligible to donate due to risk factors—and thus need a PrEP but not using the drug—should be a concern\ for both personal and public health.

Limitations of this study include the sensitive LC–MS/MS method used to detect PrEP/PEP residue in plasma, which is costly and currently not applicable for large‐scale blood donation screening. It is also unlikely to be able to detect intermittent PrEP use if taken more than 5 days preceding donation. Detection frequencies may also have been limited through the use of plasma for testing, where half‐lives of analytes are short (~17 h). The active intracellular phosphorylated forms of the TDF and FTC are preferentially partitioned within mononuclear cells, with a prolonged elimination half‐life (~17 days). Hence, testing of whole blood might be better suited for screening purposes [31].

PrEP is available both in and outside of NHS services, which makes it difficult to monitor the information provided to users/usage or engage in any in‐depth consultation and/or follow‐ups on those who choose to purchase the drug through online pharmacies in comparison to a sexual health clinic. Donors from abroad may use injectable PrEP utilising CAB, which was not included in our screening panel. In the United States, the Food and Drug Administration has recommended a 2‐year deferral for injectable PrEP/PEP [32].

In conclusion, the FAIR framework has opened up a once exclusive and discriminatory blood donor policy for GBMSM. Monitoring of the blood donation landscape following the inclusion of more GBMSM donors concluded there to be no immediate risk to blood services as of 2023. To enhance this monitoring, the behaviour and needs of this new donor group must be considered, namely PrEP use and STI incidence. It also provides the opportunity to look back at the pre‐existing UK blood donor population through a different lens. We have found an increasing number of syphilis‐positive blood donors with and without PrEP use, coinciding with a potential increase in recent HIV infections among our blood donors. Syphilis‐positive donors who do not use PrEP may benefit from starting such a regimen, something that can be advised in post‐donation discussions. The increased number of male donors with syphilis who attempt to donate also needs actioning, and those who also use PrEP present further evidence of being high‐risk donors for other infections, as was unfortunately demonstrated in the recent HIV infections seen in four young GBMSM blood donors since 2020. With NHS schemes to continue scale‐up of PrEP availability and PrEP's solidified online presence, new and more targeted approaches in utilizing our findings to better educate UK blood donors, particularly young GBMSM, will require collaboration between healthcare providers within and beyond NHSBT, universities and LGBTQ+ charities. Such steps will be integral to improving the blood donor pool as well as the reach of sexual health services for those with identified risk.

## CONFLICT OF INTEREST STATEMENT

The authors declare no conflicts of interest.

## Data Availability

The data that support the findings of this study are available on request from the corresponding author. The data are not publicly available due to privacy or ethical restrictions.
